# A Hierarchy of Time-Scales and the Brain

**DOI:** 10.1371/journal.pcbi.1000209

**Published:** 2008-11-14

**Authors:** Stefan J. Kiebel, Jean Daunizeau, Karl J. Friston

**Affiliations:** Wellcome Trust Centre for Neuroimaging, University College London, London, United Kingdom; Indiana University, United States of America

## Abstract

In this paper, we suggest that cortical anatomy recapitulates the temporal hierarchy that is inherent in the dynamics of environmental states. Many aspects of brain function can be understood in terms of a hierarchy of temporal scales at which representations of the environment evolve. The lowest level of this hierarchy corresponds to fast fluctuations associated with sensory processing, whereas the highest levels encode slow contextual changes in the environment, under which faster representations unfold. First, we describe a mathematical model that exploits the temporal structure of fast sensory input to track the slower trajectories of their underlying causes. This model of sensory encoding or perceptual inference establishes a proof of concept that slowly changing neuronal states can encode the paths or trajectories of faster sensory states. We then review empirical evidence that suggests that a temporal hierarchy is recapitulated in the macroscopic organization of the cortex. This anatomic-temporal hierarchy provides a comprehensive framework for understanding cortical function: the specific time-scale that engages a cortical area can be inferred by its location along a rostro-caudal gradient, which reflects the anatomical distance from primary sensory areas. This is most evident in the prefrontal cortex, where complex functions can be explained as operations on representations of the environment that change slowly. The framework provides predictions about, and principled constraints on, cortical structure–function relationships, which can be tested by manipulating the time-scales of sensory input.

## Author Summary

Currently, there is no theory that explains how the large-scale organization of the human brain can be related to our environment. This is astonishing because neuroscientists generally assume that the brain represents events in our environment by decoding sensory input. Here, we propose that the brain models the entire environment as a collection of hierarchical, dynamical systems, where slower environmental changes provide the context for faster changes. We suggest that there is a simple mapping between this temporal hierarchy and the anatomical hierarchy of the brain. Our theory provides a framework for explaining a wide range of neuroscientific findings by a single principle.

## Introduction

Our brains navigate our bodies, including our sensory apparatus, through a dynamically changing environment. This is a remarkable achievement, because a specific behaviour might be optimal in the short-term, but suboptimal over longer time periods. It is even more remarkable that the brain selects among different behaviours quickly and online. Causal dynamics and structure in the environment are critical for selecting behaviour, because the brain can learn this structure to predict the future, and exploit these predictions to negotiate the environment adaptively. Ontogenetically, there is good reason to believe that the brain learns regularities in the environment from exposure to sensory input and internally generated signals [1],[2]. Similarly, over evolutionary time, one can argue that selective pressure ensures the brain has the capacity to represent environmental structure [3]–[5]. In the following, we will first review the ‘free-energy principle’ [6], which suggests that ‘adaptive agents’ like the brain, in a dynamic environment, minimize their surprise about sensory input. We will then motivate the hypothesis that the environment exhibits temporal structure, which is exploited by the brain to optimise its predictions. This optimisation transcribes temporal structure in the environment into anatomical structure, lending the brain a generic form of structure-function mapping.

For an adaptive agent, surprise means sampling unexpected input given the expectations of the agent. Mathematically, surprise or improbability is quantified by −ln *p*(*y*(*a*)|*m*), where *y*(*a*) is sensory input sampled under action *a* and *m* represents the agent. Minimizing surprise depends on the agent's expectations about its sensory input and the behaviour it chooses. If these expectations (e.g., being warm but not on fire) are consistent with survival, an agent, which minimizes free-energy, will exhibit behaviour that is adapted to its environment. If an agent did not minimize surprise, it would sooner or later encounter surprising interactions with the environment, which may compromise its structural or physiological integrity (e.g., walking into a fire). Both action and perception can be understood as trying to minimize surprise about sensory input. An agent cannot minimize surprise directly because the agent does not have full knowledge about its environment [6]. However, an agent can minimize its so-called free-energy *F*≥−ln *p*(*y*(*a*)|*m*), which is an upper bound on surprise: if an agent minimises its free-energy, it implicitly minimises surprising sensory input.

To predict extero- and interoceptive input online, an agent must entertain dynamic expectations about its input using an internal model of environmental causes and their trajectories. These models reduce high-dimensional input to a few variables or ‘causes’ in the environment. These environmental causes do not need to be physical objects but can be any quantity that predicts the agent's past and future sensory input (we use prediction here in reference to the mapping between causes and their sensory consequences; this mapping subsumes but is more than a forecast of future events). Critically, from the point of view of an agent, its body is a part of the environment. Therefore, internal models embed an agent's knowledge about how environmental dynamics, including its own movements, generate sensory input [6]. The concept of ‘internal models’ which predict future sensory input due to the agent's own action is a key element of many related theoretical accounts: for example, the ‘corollary discharge hypothesis’ [7], predictive coding [8],[9], and motor control theory [10],[11].

In general, the sensory consequences of environmental causes are mediated by dynamical systems. This necessarily induces delays in the mapping between causes and their sensory consequences. How can an agent accommodate this temporal dislocation to explain causes *after* they are expressed in the sensorium [12],[13]? In this paper, we suggest that agents model sensory input using representations or ‘concepts’ that provide temporally stable predictions about future sensory input. In this paper we will use ‘concept’ to refer to a representation of an environmental cause or state that endures for about a second or more and ‘percept’ for representations that more transient. In terms of dynamical systems, concepts could be regarded as control parameters that shape the attractor or manifold on which lower-level representations unfold. This attractor provides constraints on the expected trajectories, which enable fast dynamics to be predicted by supraordinate representations that change more slowly (see Results). This rests on the assumption that the world can be modelled as a hierarchy of autonomous dynamical systems, where the output of one system controls the motion of another's states. In principle, an agent may be able to model the evolution of environmental states over milliseconds, seconds, or much longer periods of time using generative or forward models at various time-scales. For example, speech could be decomposed at various time-scales (from fast to slow): instantaneous frequency (acoustics); spectral profiles (phonemes); phoneme sequences (lexical); lexical sequences (semantics); syntactical structure (pragmatics), and so on [14].

Predictions about sensory input at fast time-scales become imprecise when projected too far into the future. One way to deal with this uncertainty is to use concepts to guide representations at shorter time-scales. If predictions of sensory input remain veridical at a fast time-scale and action ensures these predictions are fulfilled, the agent will avoid surprising input. The ensuing behaviour would be consistent with the agent's concepts. Note that an agent following this principle can still handle novel, unexpected input, although the agent might experience a large prediction error and adapt its internal model accordingly (see simulations). If the high-level representations or concepts prove correct in predicting sensory input, they confirm the validity of those concepts. Therefore, concepts can be seen as self-fulfilling prophecies, which, given a compliant environment, would appear to mediate goals, plans and long-term strategies for exchange with the world [15]. Conflict among competing explanations (i.e., concepts) for sensory data has to be resolved to avoid surprise. This conflict can be between similar time-scales; e.g. between the visual and auditory stream when experiencing the McGurk effect [16]. Conflict could also exist between different time-scales; e.g., between eating a chocolate cake or maintaining a strict diet. In robotics and motor control theory, conflict resolution among different time-scales has been addressed using hierarchical control structures [17]–[22]. These hierarchies are ordered according to the temporal scales of representations, where the slowest time-scale is at the top (c.f., ‘slow feature analysis’ [23],[24]). A hierarchical model enables a selection of predictions that is accountable to all time-scales, such that concepts and percepts are nested and internally consistent.

The novel contribution of this paper is to consider hierarchical models, in which high-level states change more slowly than low-level states, and to relate these models to structure-function relationships in the brain. The basic idea is that temporal hierarchies in the environment are transcribed into anatomical hierarchies in the brain; high-level cortical areas encode slowly changing contextual states of the world, while low-level areas encode fast trajectories. We will present two arguments in support of this hypothesis. First, using simulations, we will demonstrate that hierarchical dependencies among dynamics in the environment can be exploited to recognise the causes of sensory input. The ensuing recognition models have a hierarchical structure that is reminiscent of cortical hierarchies in the brain. Second, we will consider neuroscientific evidence that suggests the cortical organisation recapitulates hierarchical dependencies among environmental dynamics.

Note that this paper is not about hierarchies of neuronal dynamics; see e.g. [25]–[27]. Rather, we consider neuronal dynamics under hierarchical models of the environment, which, according to the principles outline above, should be represented in the brain to predict sensory input.

## Methods

In this section, we present a modelling approach to show, as a proof-of-principle, that perception can be understood in terms of inverting hierarchical models and that these models entail a separation of temporal scales.

### A Model of Perceptual Inference

Here, we model the neuronal states of an internal model in an abstract fashion, to describe their evolution under continuous sensory input. This allows us to focus on how the brain could exploit dependencies between dynamics at different time-scales, using internal models.

We pursue the notion that synthetic agents can extract information about another agent, at various time-scales, by modelling the sensory input, originating from the other agent, with an internal, generative model. We will describe how an agent produces a song and how another agent decodes the auditory input. We will deal with environmental dynamics at two different time-scales (fast and slow). In our model, we let the dynamics at the slow-scale enter as ‘control’ parameters of dynamics at the fast scale.

Our example uses birdsong: There is a large body of theoretical and experimental evidence that birdsongs are generated by dynamic, nonlinear and hierarchical systems [28]–[31]. Birdsong contains information that other birds use for decoding information about the singing (usually male) bird. It is unclear which features birds use to extract this information; however, whatever these features are, they are embedded in the song, at different time-scales. For example, at a long time-scale, another bird might simply measure how long a bird has been singing, which might belie the bird's fitness. At short time-scales, the amplitude and frequency spectrum of the song might reflect the bird's strength and size.

It may be that the recognition of human song or speech is implemented using hierarchical structures too; although the experimental evidence for this seems much scarcer. In particular, speech has been construed as the output of a multi-level hierarchical system, which must be decoded at different time-scales [32],[33]. For example, while a spoken sentence might only last for seconds, it also conveys information about the speaker's intent (an important environmental cause) at much longer time-scales. Here we use the avian example to provide a proof-of-principle of a commonplace and generic mechanism: to communicate via audition, both birds and humans need to embed information, at various time-scales, into sound-waves at a fast time-scale and the recipient must invert a dynamic model to recover this information. Our objective is to show that such communication can be implemented using hierarchical models with separation of temporal scales. In the following, we describe a two-level system that can generate sonograms of synthetic birdsong and model the perception of this song. Similar systems, using a single generating oscillator, have been proposed to generate birdsong [34]. What we want to show is how another (synthetic) bird can use a heard song to extract information about the (synthetic) singing bird, using at least two separable temporal scales.

### A Generative Birdsong Model

Recently, Laje et al. [34] generated synthetic birdsong by modelling the bird's vocal organ using a variant of the van der Pol oscillator. Furthermore, Laje and Mindlin [35] introduced variations in their bird-song generator by adding a second level, which acts as a central pattern generator (CPG) driving the van der Pol oscillator. This hierarchical, two-level model can produce different songs, depending on the driving input and parameters of the CPG. In our model, we use this principle of letting a slow CPG drive a faster system that produces song syllables. However, for simplicity, when decoding the produced song, we model the sonogram; i.e. the time-frequency representation of birdsong, instead of the acoustic time-series. Although this renders our model phenomenological with respect to dynamics in the vocal organ, it allows us to focus on the interaction between the first-level (vocal organ) and the second-level (central pattern generator). It would be straightforward (but computationally expensive) to make the first level a generative model and decode the temporally resolved time-series.

To generate birdsong sonograms, we use the Lorenz attractor, for both levels.
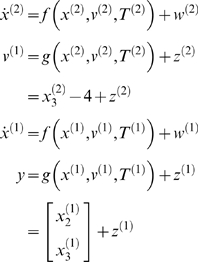
(1)where, in general, *v* ^(*i*)^ represent inputs to level *i* (or outputs from level *i*+1), which perturb the possibly autonomous dynamics among that level's states *x* ^(*i*)^. The nonlinear function *f* encodes the equations of motion of the Lorenz attractor:
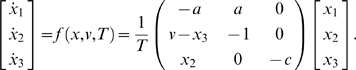
(2)


For both levels, we used *a* = 10 (the Prandtl number) and *c* = 8/3. The parameter *T* controls the speed at which the Lorenz attractor evolves; here we used *T* ^(1)^ = 0.25*s* and *T* ^(2)^ = 2*s* so that the dynamics at the second level are an order of magnitude slower than at the first. At the second-level we used a Rayleigh number; *ν* ^(2)^ = 32. We coupled the fast to the slow system by making the output of the slow system 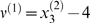 the Rayleigh number of the fast. The Rayleigh number is effectively a control parameter that determines whether the autonomous dynamics supported by the attractor are fixed point, quasi-periodic or chaotic (the famous butterfly shaped attractor). The signals generated are denoted by *y*, which comprises the second and third state of *x* ^(1)^ (Equation 1).

**Figure 1:**
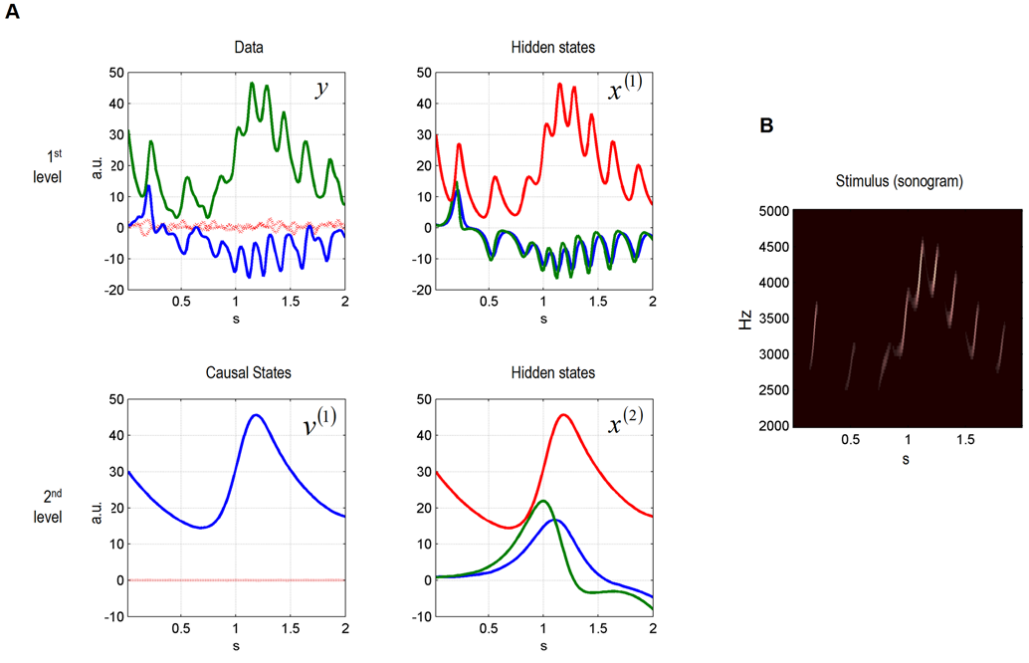
Data and states, over two seconds, generated by a two-level birdsong model. (A) At the first level, there are two outputs (i.e., data) (left: blue and green solid line) and three hidden states of a Lorenz attractor (right: blue, green, and red solid line). The second level is also a Lorenz attractor that evolves at a time-scale that is one magnitude slower than the first. At the second level, the causal state (left: blue solid line) serves as control parameter (Rayleigh number) of the first-level attractor, and is governed by the hidden states at the second level (right: blue, green, and red solid line). The red dotted lines (top left) indicate the observation error on the output. (B) Sonogram (time-frequency representation) constructed from model output. High intensities represent time-frequency locations with greater power.

We will call the vectors *x* ^(*i*)^ ‘hidden’ states, and the scalar *v* ^(1)^ the ‘causal’ state, where superscripts indicate model level and subscripts refer to elements. At each level we modelled Gaussian noise on the causes and states (*w* ^(*i*)^ and *z* ^(*i*)^) with a log-precision (inverse variance), of eight (except for observation noise *z* ^(1)^, which was unity). We constructed the sonogram (describing the amplitude and frequency of the birdsong) by making |*y*_1_| the amplitude and *y*_2_ the frequency (scaled to cover a spectrum between two and five kHz). Acoustic time-series (which can be played) are constructed by an inverse windowed Fourier transform. An example of the system's dynamics and the ensuing sonogram are shown in Figure 1A and 1B. The software producing these dynamics, the sonogram and playing the song can be downloaded as Matlab 7.4 (Mathworks) code (see software note). The synthetic birdsong passes as birdsong-like. This model can be regarded as a generative or forward model that maps states of the singing bird to sensory consequences (i.e., the sonogram).

Inversion of this forward model corresponds to perception or mapping from the sonogram to the underlying cause in the singing bird. In this example, recognition involves the online estimation of the states at both levels. Although two of the states (those controlling amplitude and frequency of the acoustic input) at the first-level are accessed easily, the third 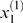 is completely hidden. It is important to estimate this state correctly because it determines the dynamics of the others (see Equation 2). Model inversion also allows the listening bird to recognise the slowly varying states at the second level, *x* ^(2)^ (c.f., the syntax of the chirps), which cannot be heard directly but must be inferred from the fast sensory input. This inversion problem is difficult to solve because the bird can only infer states at both levels through the nonlinear dynamics of the Lorenz attractor. In the following, we will sketch a variational scheme to show how inversion of a stochastic nonlinear hierarchical model can be implemented. A detailed description of this inversion is beyond the scope of this paper. However, the details and conceptual background of the approach can be found in [36].

### Variational Inversion

Given some sensory data *y*, the general inference problem is to compute the marginal likelihood of the data, given a model *m* of the environment:
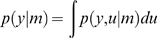
(3)where the generative model *p*(*y*,*u*|*m*) = *p*(*y*|*u*,*m*)*p*(*u*|*m*) is defined in terms of a likelihood *p*(*y*|*u*,*m*) and prior *p*(*u*|*m*) on the model's states. In Equation 3, the states *u* = {*x*,*v*} subsume the hidden and causal states at all levels. The model evidence can be estimated by converting this difficult integration problem (Equation 3) into an easier optimization problem by optimising a free-energy bound on the log-evidence [37]. This bound is constructed using Jensen's inequality and is a function of an arbitrary *ensemble* density, *q*(*u*):
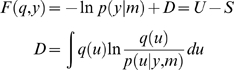
(4)


The free-energy comprises an energy term *U* = −〈ln *p*(*y*|*u*)+ln *p*(*u*)〉*_q_* and an entropy term *S* = −〈ln *q*(*u*)〉*_q_*. It is defined uniquely, given the generative model *m* and is an upper bound on the surprise or negative log-evidence because the Kullback–Leibler cross-entropy or divergence *D*, between the ensemble and exact conditional density, is always positive. Minimising the free-energy minimises the divergence, rendering the ensemble density *q*(*u*)≈*p*(*u*|*y*,*m*) an approximate posterior or conditional density. When using this approach for model inversion, one usually employs fixed-form approximations of the conditional, which takes a simpler parameterized form *q*(*u*|*λ*) [36]. Variational learning optimizes the free-energy with respect to the variational parameters *λ*; i.e., the sufficient statistics of the approximate conditional:
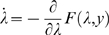
(5)


Generally, the variables *λ* correspond to the conditional moments (e.g., expectation and variance) of the states. A recognition system that minimizes its free-energy efficiently will therefore come to represent the environmental dynamics in terms of moments of the conditional density, e.g., the conditional expectations and variances of *q*(*u*|*λ*) = *N*(*μ*,Σ): *λ* = {*μ*,Σ}. We assume that the conditional moments are encoded by neuronal activity, i.e., Equation 5 prescribes neuronal dynamics. These dynamics implement Bayesian inversion of the generative model, under the variational approximations entailed by the form of the ensemble density. In practice, Equation 5 is implemented using a message passing scheme, which, in the context of hierarchical models, involves passing prediction errors from one level up to the next and passing predictions down, from one level to the next. The prediction errors are simply the difference between the causal states at any level and their prediction from the level above, evaluated at the conditional expectations [6],[8]. This means that we have two sets of neuronal populations, one encoding the conditional expectations of states of the world and another encoding prediction error. The dynamics of the first are given by Equation 5, which can be formulated as a simple function of prediction error; *ε* ^(*i*)^ = *v* ^(*i*)^−*g*(*x* ^(*i*+1)^,*v* ^(*i*+1)^,*T* ^(*i*+1)^), which is the activity of the second population. See [6],[8] and [36] for details.

Here, Equations 1 and 2 specify the generative model in terms of the likelihood function *p*(*y*|*u*,*m*), which follows from Gaussian assumptions about the random terms. The hierarchical form of the model induces empirical ‘structural’ priors, which provides top-down constraints on the evolution of states generating sensory data. In addition to these structural priors, there are also empirical priors on the temporal evolution of the states that derive from modelling states in generalised coordinates of motion:

### Generalised Coordinates of Motion

Under the free-energy principle, the agent must implement models that represent, at each moment in time, the dynamics of causes in the environment, as in Equations 1 and 2. Because these equations also prescribe how the motions of various states couple to each other, our generative model covers not just the states but their motion, acceleration, and higher order velocities. These are referred to collectively as ‘generalised coordinates of motion’, in the sense that the trajectory (or motion) of any dynamical system can be described within this frame of reference. We use the following notation for a vector of generalized coordinates: *ũ* = {*u*,*u*′,*u*″,*u*‴…}, whose entries are the current state *u* (Equation 3), its motion and higher order temporal derivatives. This frame of reference can be thought of encoding the trajectory at any instant, in terms of the coefficients of the polynomial expansion in time:
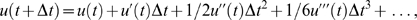
(6)where Δ*t* is an arbitrary time interval. Equation 6 is the Taylor series of the trajectory as a function of time. Therefore, specifying the generalized coordinates of motion at any time point encodes the present, past and future states of the system [38]. This representation is related to the notion of ‘spatiotemporal receptive fields’ that describe the response of neurons to certain spatiotemporal dynamics in the environment [39], see also [40]. The sufficient statistics *λ* (Equation 5) of the conditional generalized motion *q*(*ũ*|*λ*) encodes trajectories in a probabilistic fashion. Uncertainty on each generalised coordinate controls how far into the future the trajectory can be specified with confidence (for example, to represent a smooth trajectory that extends far into the future, one needs high precision on high-order derivatives). In other words, from the agent's perspective, the precision of both its memory and its prediction of sensory input will fall with distance from the current time as a function of the conditional precision of its state in generalized coordinates. The empirical priors that obtain from modelling in generalised coordinates ensure smooth continuous estimates of trajectories and enable online inversion. For more details please see [36].

In our simulations, we used six high-order temporal derivatives for the hidden states *x* ^(1)^ and *x* ^(2)^, and two for the causal state *v* ^(1)^. It should be noted that although generalised coordinates finesse the recognition dynamics prescribed by Equation 5, the focus of this work is on the empirical priors that are conferred by the hierarchical structure of the model. It is these that enable the separation of temporal scales and prediction over long time-scales. The routines (incl. Matlab source code) implementing this dynamic inversion and the birdsong example are available as academic freeware (Statistical Parametric Mapping package (SPM8) from http://www.fil.ion.ucl.ac.uk/spm/; Dynamic Expectation Maximization (DEM) Toolbox).

## Results

### Simulations of Birdsong Perception

In this section, we generate synthetic birdsong using the coupled Lorenz oscillators described above and model a ‘listening’ bird during song recognition by inverting the model using Equation 5, where we consider the conditional moments, *λ* of *q*(*ũ*|*λ*) to be encoded by neuronal activity (under the Laplace approximation we need only encode the conditional expectation because the conditional covariance is an analytic function of the expectation [38]). The conditional expectation of the hidden states at the first level encodes fast auditory input, whereas the conditional expectation at the second level encodes slowly varying states that engender changes in the first-level's attractor manifold, through the causal state that links levels.

**Figure 2:**
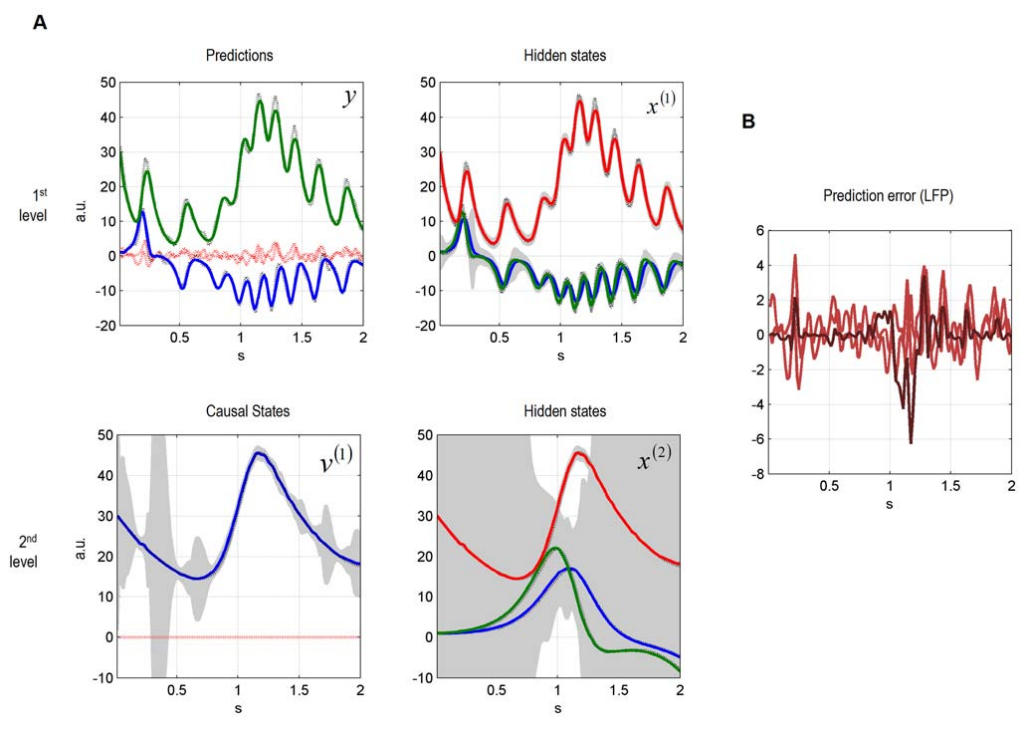
Dynamic online inversion of the data presented in Figure 1. Observed data (see Figure 1) are now shown as black, dotted lines, and the model predictions as solid, coloured lines. (A) The 90% confidence interval around the conditional means is shown in grey. The prediction error (i.e., difference between observation and model prediction) is indicated by red dotted lines. (B) Simulated local field potentials (LFPs) caused by the prediction error time series of both levels. See text for their simulation. Red: LFPs at first level, dark red: LFP at second level.

In Figure 1A we plot the hidden states, cause and sensory products for the synthetic bird-song generation. One can see immediately that the two levels have different time-scales due to their different rate constants (Equations 1 and 2). The resulting sonogram is shown in Figure 1B. The results of the online inversion (i.e., song perception) are shown in Figure 2A. At the first level, the uncertainty about the states was small, as indicated by narrow 90% confidence intervals, shown in grey. At the second-level, the system tracks the hidden and causal states veridically. However, as these variables are inferred through the sensory data, the uncertainty about the hidden state reaches, intermittently, high values. The uncertainty about the hidden states at the second-level is very high, because these variables can only be inferred via the causal state *v* ^(1)^. What would these dynamics look like if one recorded electrophysiological data from the corresponding neuronal populations? In Figure 2B, we plot simulated local field potentials (LFP) for both levels.

To simulate the LFPs we multiplied the prediction errors by their precision to simulate the activity of neurons encoding prediction error: We assume here that LFPs are an expression of prediction error, see [8] and text following Equation 5. The prediction error of all states is relatively low, showing transient variations that are used to adjust the conditional estimates of the model's states (Figure 2B). In summary, these results show that the model can not only generate birdsong dynamics but, using the free-energy principle, it can be used to decode incoming sensory input with relatively high precision. Critically, at the second level, the decoding (listening) bird infers hidden states that evolve slowly over time. This is an important result because the values of the states at the second level specify the attractor manifold, and therefore the trajectory of states at the first. In other words, one location in state space at the higher level specifies a sequence of states at the lower. Because we have inferred or decoded the motion of states at the second level the synthetic bird has effectively recognised a sequence of sequences. In principle, by adding a further level the bird could represent sequences of sequences of sequences and so on to elaborate high-level concepts about what is happening in the environment.

We deliberately chose to generate both levels of the birdsong with the same (Lorenz) attractor to show it is possible to invert generative models with temporal hierarchies comprising more than two levels: because we were able to reconstruct the dynamics at the second level given the first, we can argue, by induction, that this process is repeatable to any hierarchical order, with increasing temporal scales. This is because the dynamics at the second level are exactly the same as the first (but evolve more slowly). Having established that the online perception returns sensible results, we can ask two interesting questions. First, what happens when the sensory input violates hierarchical predictions? Second, how would the second level express itself empirically, using LFPs and lesion studies?

### Surprising Songs

**Figure 3:**
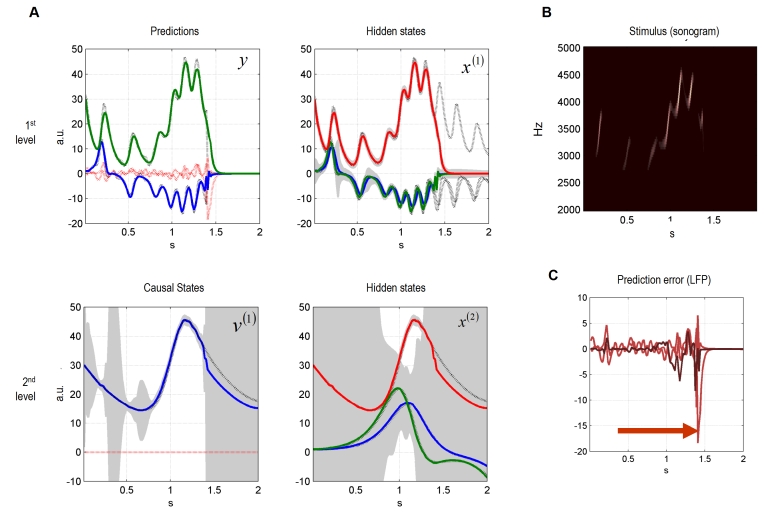
Dynamic online inversion of surprising input. The sensory data presented in Figure 1 were set to zero at 1.4 seconds, see also Figure 2. (A) The first-level dynamics return to zero after a transition period of ca. 100 ms. We plotted the hidden states and the causal state as dotted lines, for the uninterrupted song. The second-level increases its conditional uncertainty and no longer constrains the first-level dynamics. (B) Sonogram constructed from output. (C) Simulated LFPs of both levels. The red arrow indicates time point of largest prediction error due to interruption.

First, we simulated a surprising song, in which the last chirps were omitted. We stopped the bird's singing after 1.4 seconds, which effectively removes the last two chirps (Figure 3A and 3B). The recognition system, at the first level, correctly predicts zero amplitude auditory input, after the interruption. However, this does not happen immediately but after a short period of about 100 ms. At the second level, the uncertainty about the cause increases massively and maintains its trajectory, following the expected sequence of chirps. At both levels, for a brief period after the interruption, there is a large prediction error (Figure 3C). In summary, the system's response shows that both levels work together to explain the unexpected cessation of sensory input. While first-level dynamics suppress prediction error by fitting sensory data, the second-level representations increase their uncertainty.

This example was chosen to show how hierarchical models might disclose themselves empirically. Consider the simulated LFP responses based on prediction error in Figure 3C. The marked responses at the premature termination of the song (red arrow) can only be explained by a violation of predictions (surprise) over time. This is because we have simulated an evoked response to the *omission of a stimulus*. In the absence of predictions, a stimulus that is not there cannot elicit any response. The hierarchical nature of these predictions derives from two aspects of the model. The dynamical hierarchy, encoded by the generalised motion within each level, and the structural hierarchy entailed by the two-levels. In the next simulation, we examine their relative contribution to omission-related prediction error responses by removing the second level. We hoped to show that the omission response was attenuated because the prediction from the slower temporal scale was no longer available.

### A Synthetic Lesion Study

**Figure 4:**
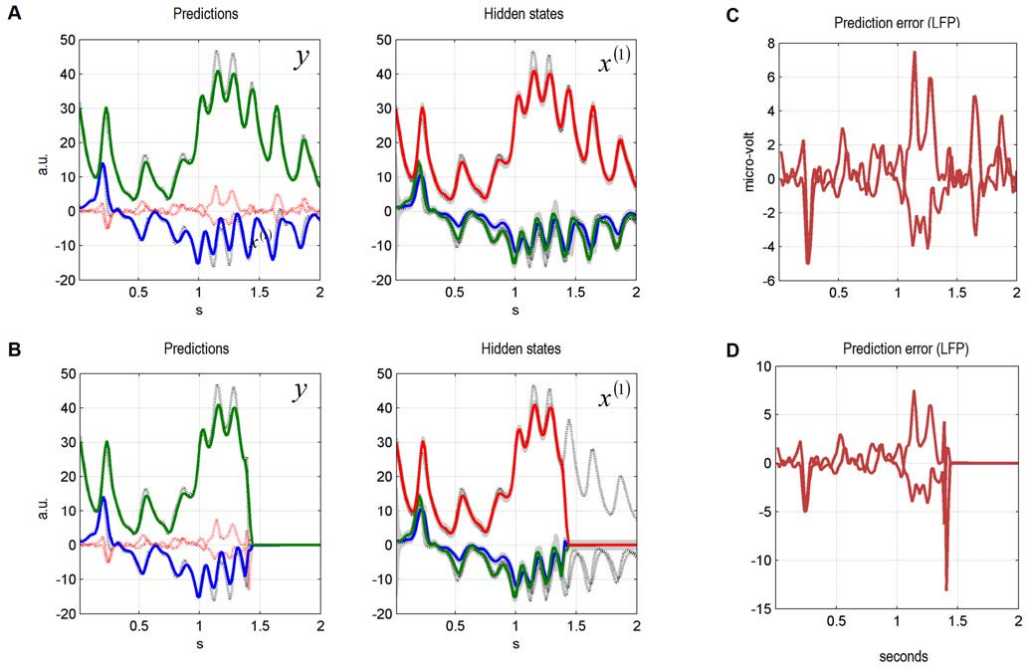
Single-level model dynamic online inversion of the data presented inFigures 1 and 3. (A) The single-level model can explain the data (no song interruption) well. (B) The single-level model quickly approaches the zero line after an interruption at 1.4 seconds. (C) Simulated LFPs for model inversion in (A). (D) Simulated LFPs for model inversion in (B).

Here we simulated a synthetic bird whose second level had been removed. In Figure 4A, we show the inversion of the ensuing single-level model using the same data as above. The prediction error at the first level in Figure 4C is greater than for the two-level system (Figure 2B). This is expected because the single-level model is not informed about the slowly changing parameter from the second-level attractor. In other words, the two-level system attains a lower prediction error because it can model slow environmental dynamics, which results in a better description of sensory input.

**Figure 5:**
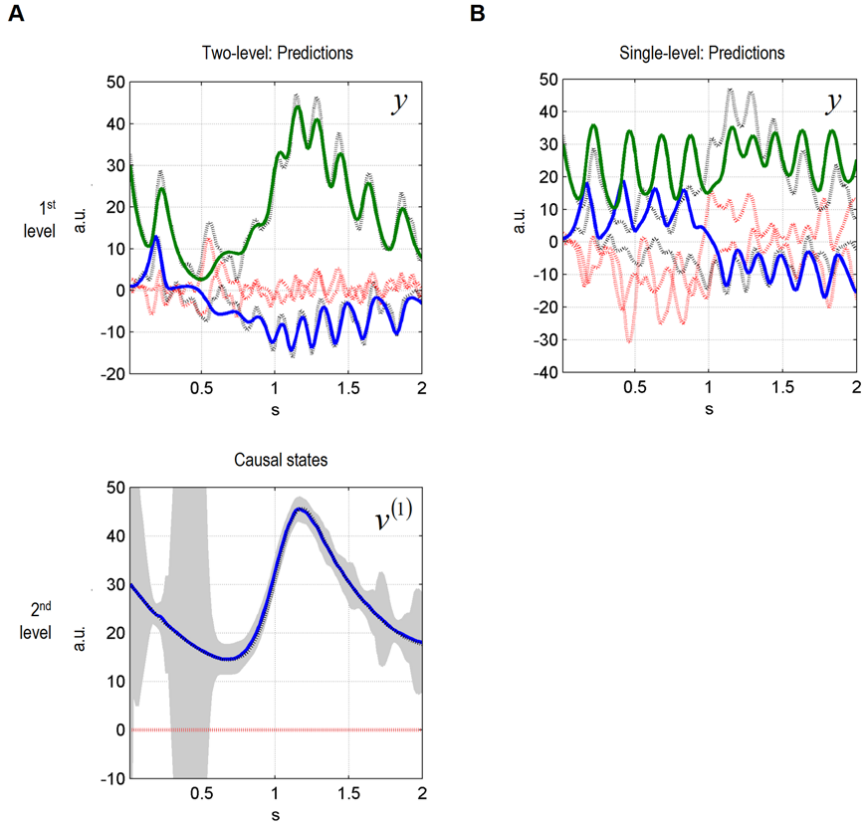
Comparison of single- and two-level model inversion of high-noise birdsong data. We show only the output of each model and the causal state of the two-level model. (A) The two-level model can explain the data relatively well, although it misses the third syllable. (B) The single-level model is unable to predict the data at all.

Figure 4B shows what happens when the song stops prematurely. As predicted, the omission response of the single-level system is smaller than for the two-level system and reaches zero more quickly (Figure 4D). This means that the two-level system is less forgiving of violations in long-term temporal structure, when predicting sensory input. This is an important result because it means that, given unexpected input, the two-level model produces a larger prediction error than the simpler single-level model. Usually, models that produce smaller prediction errors are better than models that produce larger prediction errors. In other words, if our task were to model interrupted birdsongs, the two-level model is worse than the single-level model. The critical point is that although this behaviour can be framed as a disadvantage from a modelling perspective, it entails several advantages for the agent:

First, the larger and more enduring prediction error of the two-level system signals that something unexpected and potentially important has happened (a cat might have put an abrupt end to the rendition). The second-level prediction error could then be explained away by supraordinate causes (i.e., a nearby predator) whose representation may be essential for survival. In short, hierarchical systems can register and explain away surprising violations of temporal succession, on extended time-scales. Second, the two-level system can infer slowly changing causes to which the single-level system is blind. These second-level dynamics may carry useful information; for example, that the singing bird is strong and well-fed. Missing this information may pose a serious disadvantage when it comes to choosing a mate. Finally, the second level adds stability to the inversion process and renders recognition more robust to random fluctuations in the environment. The coupling of the fast to the slow level improves inference on degraded sensory input by providing empirical priors. This is shown in Figure 5A, where we increased the noise level of the sensory input by an order of magnitude. The two-level model can cope with this level of noise (although the third syllable is missed; Figure 5A). In contrast, the single-level system fails to predict the sensory data completely (Figure 5B). This difference in recognition is due to veridical prior knowledge from the second level, which confers a more enduring prediction of sensory sequences.

A key aspect of the recognition model above rests on the nonlinearity of the internal model. It is this nonlinearity that allows high-level states to act as control parameters to reconfigure the motion of faster low-level states. If the equations of motion at each level were linear in the states, each level would simply convolve its supraordinate inputs with an impulse response function. This precludes the induction of faster dynamics because linear convolutions can only suppress or amplify the input frequencies; they cannot create new frequencies. However, the environment is nonlinear, where long-term causes may disclose themselves through their influence on the autonomous nonlinear dynamics of other systems. To predict the ensuing environmental trajectories accurately, top-down effects in the agent's internal model must be nonlinear too.

## Discussion

The simulations have shown how environmental trajectories at two different time-scales can be extracted from fast sensory input. This simple example of how a synthetic bird recognises songs provides a metaphor for how the human brain might exploit temporal structure in the environment. Obviously, the brain affords many more levels than two and operates on much higher-dimensional input. However, the principle of hierarchical inference, with separation of time-scales, could be an inherent part of neuronal computations. If the generative model employed by the brain embodies autonomous dynamics that are coupled nonlinearly by control parameters, each level in the hierarchy may represent a specific time-scale. In the following, we will discuss two bodies of neuroscientific evidence for such a mapping: (i) modulatory backward connections which operate at slower time-scales than forward connections and (ii) a cortical gradient of environmental time-scales. We then relate the principle of hierarchical inference to other theoretical accounts in neuroscience.

### Neuroscience Account

#### Modulatory backward connections

There is extensive literature on the hierarchical organisation of the brain, in particular of the cortex [41]–[44]. This organisation has been studied most thoroughly in the visual system, where cortical areas are regarded as forming a hierarchy; with lower areas being closer to sensory input. The notion of a hierarchy rests upon the distinction between forward and backward connections [41], [45]–[48]. This distinction is based on the specificity of the cortical layers that are the predominant sources and origins of extrinsic connections in the brain. Forward connections arise largely in superficial pyramidal cells, in supra-granular layers and terminate in spiny stellate cells of layer four or the granular layer of a higher cortical area [41],[49]. Conversely, backward connections arise largely from deep pyramidal cells in infra-granular layers and target cells in the infra- and supra-granular layers of lower cortical areas. Intrinsic connections are both intra- and inter-laminar and mediate lateral interactions between neurons that are a few millimetres away. Due to convergence and divergence of extrinsic forward and backward connections, low visual levels like the primary visual cortex (V1) have small spatial receptive fields, whereas higher visual areas have larger receptive fields; e.g., lateral-occipital cortex [50].

There is a key functional distinction between forward and backward connections that renders backward connections more nonlinear or modulatory in their effects on neuronal responses, e.g., [48]. This is consistent with the deployment of voltage sensitive and non-linear NMDA receptors in the supra-granular layers that are targeted by backward connections. Typically, the synaptic dynamics of backward connections have slower time constants [51]. This has led to the notion that forward connections are driving and elicit an obligatory response in higher levels, whereas backward connections have modulatory effects and operate over greater spatial and temporal scales. This is crucial, because modulatory influence, from a higher level, with slow time constants, suggests that information from the higher level provides contextual information to the lower level. These experimental findings are matched by our theoretical account. In our simulations, evidence for a slow modulatory influence on a lower level can be observed in Figure 3A. Here, contextual top-down influence during online inversion prevents the first-level from reacting quickly to a surprising (unlikely) change in the sensory input. It takes a relatively long period (100 ms) before the dynamic inversion recognizes the unexpected end of the song. This slow transition to a new input regime is due solely to the slow contextual influence of the second level; the transition is much faster (∼10 ms) when one removes the contextual influence (Figure 4B). Note that we did not tune the inversion algorithm to ensure higher levels provide slow contextual guidance for lower levels. Rather, the generative model of a temporal hierarchy enforces that hierarchical separation of temporal scales.

#### Rostro-caudal gradient of environmental time-scales

Assuming that the brain employs a temporal hierarchy and that ‘wiring costs’ [52] among levels are minimised, one might expect (i) that low levels of the cortical hierarchy are anatomically close to primary sensory areas and (ii) that the juxtaposition of time-scales (fast to slow) is conserved, when mapped to hierarchically disposed cortical areas. Indeed, systems neuroscience provides experimental evidence that there is a rostro-caudal gradient in cortex, along which the time-scales of representations generally increase, from fast (caudal) to slow (rostral). In Table 1, we list brain areas/systems for which we review the evidence that these form levels in an anatomic-temporal hierarchy in supporting material (Text S1). The time-scales of environmental dynamics in Table 1 are rough estimates based on this review. In this picture, cortico-cortical long-range connections allow for coupling among time-scales. Note that although the view presented in this paper is entirely cortico-centric, we speculate that a cortical anatomic-temporal hierarchy is also expressed in subcortical structures.

**Table 1:** Brain areas and systems for which we review evidence (Text S1) that cortical structure–function relationships follow a rostro-caudal gradient.

Cortical Areas	Brief Description	Time-Scale of Environmental Dynamics	Section in Text S1
Sensory and association cortex	Sensory processing follows a temporal hierarchy	Milliseconds to hundreds of milliseconds	Section 1
Primary motor and premotor cortex	Motor areas serve the hierarchical prediction of the sensory consequences of movement trajectories	Tens of milliseconds to seconds	Section 2
Rostral anterior cingulate cortex	Hierarchical, contextual influence on action prediction	Tens of seconds to much longer periods	Section 3
Lateral prefrontal cortex	Hierarchically ordered ‘cognitive control’ system	Tens of seconds to much longer periods	Section 4
Orbitofrontal cortex	Representation of temporally most stable environmental states	Very long periods	Section 5

The location along this gradient determines the time-scale of the environmental dynamics that are represented.

#### Links to other theoretical accounts

The concept of modelling sensory dynamics and their relation to neuronal representations can be related to several approaches in theoretical physics [53]–[59]. The most important is ‘synergetics’ described in Haken [56], where Jirsa and Haken [58] further elaborated the theory to relate it to electromagnetic observations of brain activity. Synergetics embodies the principle that fast dynamics are ‘enslaved’ by slow dynamics, governed by a few ‘order parameters’ naturally incorporating time-scale separation. Synergetics has been demonstrated in behavioural dynamics like bimanual coordination, where the dynamics of finger movements are modelled in terms of fast and slow dynamics. As shown in [59], this framework can be used to analyze brain dynamics as measured with magnetoencephalography. In [57], the synergetics approach was employed to model the recognition of behavioural patterns like arm movements. The principle of a temporal hierarchy might also be linked to accounts of environmental or neuronal multi-scale dynamics, e.g., [53],[54]. In another related approach from theoretical physics, it has been shown that, under certain constraints, coupled nonlinear systems can transfer information from fast to slow time-scales [55].

There is extensive literature on the hierarchical structure of human behaviour, see [60] for a recent example and [61],[62]. In [63], Botvinick proposed a hierarchical model of behavioural sequences, using recurrent neural networks, where high levels in a hierarchy encode slow time-scales, while low levels encode fast input/output. The temporal hierarchy emerged, after learning, without imposing specific constraints. This is an important result, that is shared with several accounts in the robotics literature, where a hierarchy of time-scales in recurrent networks emerges naturally from optimizing a robot to perform navigation tasks [21],[22],[64],[65].

There are several theories that relate to the hypothesis that the operations of specific brain systems pertain to temporal structure of the environment. An exemplary approach is Fuster's sensorimotor hierarchy [12],[66],[67]. Fuster postulates that prefrontal cortex integrates behaviour (motor) over time, while interacting with posterior (sensory) cortical areas. This theory rests on two interacting hierarchies (see Figures 1and 2 in [66]). In spirit, this model is close to what we have formulated. However, one conceptual difference is that we regard the whole of cortex as a single hierarchy. In our model, the unifying feature of the hierarchy is a rostro-caudal gradient of time-scales. Fuster derives the need for two sub-hierarchies from the division of motor and perceptual resources. We believe that this division might prove unnecessary because, according to the free-energy principle, the brain has the singular task of predicting sensory input. This means that the generators of motor output simply predict sensory consequences of anticipated [intended] movements, e.g., [40],[68].

Other models, in particular from motor control theory, try to explain perception and action via forward modelling and reinforcement learning, e.g., [17],[69]. There are several important differences, between these accounts and the approach used above. Our approach uses an explicit separation of time-scales. Another key difference lies in the generality of our inversion algorithm, with nonlinear evolution and output functions at each level (recurrent networks often use linear mixing of the input and a sigmoid output nonlinearity). In addition, our algorithm enables inference on the state precisions such that dynamic uncertainty is quantified. This is probably important for an adaptive agent because behaviour should not only depend on some estimated state of the environment but also on the agent's uncertainty about these estimates. Other differences exist at a more technical level: we use a variational Bayesian framework in generalised coordinates, which enhances the stability and simplicity of the online inversion scheme [36].

There is a large experimental and theoretical literature on coupled neuronal dynamics, e.g., [25],[26],[54],[70], which is distinct from the current treatment. The neuronal dynamics considered in this work are determined by the free-energy principle (Equation 5). This means that any separation of temporal scales emerges explicitly from the generative model which is transcribed from the environment. This separation is not an inherent property of coupled neuronal systems *per se*. One important implication is that neuronal dynamics themselves may not relate directly to dynamics of sensory input but rather to the inversion scheme used to optimise the model of that input. However, it is interesting to note that there are reports of a simple relationship between the temporal aspects of sensory input and neuronal dynamics, particularly in the auditory domain [70],[71].

### Conclusion

We have proposed that the brain employs a hierarchical model, where nonlinear coupling among hierarchical levels endows each with a distinct temporal scale. At low levels of this hierarchy; e.g., close to primary sensory areas, neuronal states represent the trajectories of short-lived environmental causes. Conversely, high levels represent the context in which lower levels unfold. Critically, at each level, representations depend on, and interact with, representations at other levels. We presented simulations that provide a proof of concept that a temporal hierarchy is a natural model to recover information about dynamic environmental causes. In addition, we have discussed empirical findings, which support the conclusion that cortical structure recapitulates a hierarchy of temporal scales.

The principle of a temporal hierarchy provides a theoretical framework for experiments in systems neuroscience. The predictions based on this account could be addressed by making time-scale an experimental factor. For visual areas, Hasson et al. [72] provide a compelling example of such paradigms.

## Supporting Information

Text S1 Review of neuroscientific evidence. In sections 1 to 5, evidence is reviewed that cortical structure and function reflect an anatomic-temporal hierarchy, following a rostro-caudal gradient.
(0.13 MB PDF)
