# Supplementary material for: A Hierarchy of Time-Scales and the Brain
Source: PLoS Comput Biol. 2008 Nov 14;4(11):e1000209. doi: 10.1371/journal.pcbi.1000209 (PMC2568860; doi:10.1371/journal.pcbi.1000209)
Supplement: Text S1 — Review of neuroscientific evidence. In sections 1 to 5, evidence is reviewed that cortical structure and function reflect an anatomic-temporal hierarchy, following a rostro-caudal gradient. (0.13 MB PDF) [file pcbi.1000209.s001.pdf]

Section 1

Sensory and association cortex

Visual system

In systems neuroscience, two visual processing streams have been described, the ventral (occipital/temporal) and dorsal (occipital/parietal) stream [1,2]. The ventral stream has been shown to be involved in object and person recognition [3]. The dorsal stream is thought to be involved in supplying visual parameters for planning or executing actions [4,5]. For the visual ventral stream, it is well-established that there is a spatial hierarchy which progresses from occipital cortex towards inferior temporal areas [6]. At low levels of this hierarchy, simple visual features are processed, whereas higher levels are concerned with complex configurations of visual inputs like faces and objects [7]. Visual input is rarely static; i.e., the visual scene can change quickly due to saccadic and micro-saccadic eye movements, or moving objects. Therefore, small and 'simple' features like contrast differences and edges are in constant motion, in retinotopic space. In other words, at low levels of the visual hierarchy, from the perspective of neuronal populations, visual features are transient. For example, there is evidence for temporal frequency-specific maps in primate and human primary visual cortex [8,9]. Furthermore, Hasson et al. [10] have shown that visual areas are differentially responsive to the temporal scales of visual dynamics, in a hierarchical fashion, following a rostro-caudal gradient from primary visual cortex to extra-striate cortex and higher cortical areas.

When ascending the hierarchy, classical receptive fields become larger and the temporal dynamics of spatially extended features become more protracted. This means that the hierarchy in the ventral stream can be described as spatiotemporal; see also [11]. This line of thought is supported by findings which shows that 'mid-level' visual areas in the visual ventral stream, the lateral-occipital complex (LOC), process motion-invariant features of rotating objects [12]. To do this LOC must have integrated visual information over space and, to attain motion-invariance, over time.

Recently, Konen and Kastner [13] provided evidence for a hierarchy in the dorsal stream; i.e., high levels in parietal cortex represent invariant object properties. Critically, part of the dorsal stream is assumed to represent movement-relevant sensory (e.g. visual) trajectories [4]. To extract invariant information, high levels of the dorsal stream must integrate over 'fast' features inferred by lower levels.

Support for a similar spatiotemporal hierarchy is presented in [14], which describes a hierarchical model for recognition of biological motion, involving activity in superior temporal sulcus (STS). The authors motivate their model using spatial considerations, but the computational operations (like temporal integration) make it a spatiotemporal model. Furthermore, a temporal integration model was used to show that activity in lateral intraparietal area (LIP) can be interpreted as part of a decision making process, about dynamic sensory input [15]. In summary, in a hierarchical model, one could consider both the visual dorsal and ventral streams as parallel and interacting spatiotemporal sub-hierarchies [13,16,17].

Auditory system

In the auditory system, a temporal, hierarchical organisation is widely accepted. Auditory information is highly structured in the temporal domain [18–20] and much research in this area tries to identify anatomical hierarchies pertaining to the different temporal scales of auditory input; e.g. [21,22]. Several authors have postulated the existence of auditory dorsal and ventral streams, which appear to pertain to 'audition-for-action' (dorsal) and 'audition-for-perception' (ventral) [23–25]. A common theme of these and many other auditory studies is that temporal integration is necessary to obtain invariance with respect to features at specific time-scales. In addition, there is evidence for a hemispheric difference in the auditory time-scales processed by the lateralized auditory cortex: time-scales in the right hemisphere seem to be slower (perception of tonal patterns) than homologous time-scales of the left hemisphere (speech processing), e.g., [26–28].

Somatosensory system

For the somatosensory system (studied in rats), there are hints that a similar temporal hierarchy prevails [29]. Also, in humans, recent research shows that haptic input produces specific and predictable spatiotemporal sensory patterns: In the 'cutaneous rabbit illusion', temporal violations of predictions, i.e., deviation from a predicted haptic, spatiotemporal input pattern are exploited to generate an illusory percept [30]. The spatiotemporal illusion results in activity (as compared to appropriate control conditions) in primary somatosensory cortex, corresponding to the skin location of the illusory haptic input. This indicates a prediction error suggestive of a top-down modulation by higher level cortices coding the trajectory of the expected somatosensory input.

Section 2

Primary motor and premotor cortex

Human movements are initiated and controlled over a relatively fast time-scale; eye movements like saccades are executed on a time-scale of 20 to 200 ms, [31], whereas body and limb movements evolve over ca. a hundred to a thousand ms [32]. By using movements an agent can optimise the free-energy bound on surprise by re-sampling sensory data and reducing prediction error at the lowest levels of the hierarchy. Therefore, in a temporal hierarchy, motor units should be concerned with dynamics at the time-scale of body movements. In support of this view, there is experimental evidence that neurons in the primary motor cortex of monkeys represent complex movement trajectories and predict the velocity of hand movements up to 100 ms into the future [33,34].

Dorsal and ventral premotor areas are thought to be involved in various aspects of motor preparation, planning, execution and observation; e.g., [4,35]. Assuming a temporal hierarchy, premotor areas would represent the trajectories of motor units at a slower time-scale than primary motor areas, probably for about a second into the future. It is also well-established that premotor areas are involved when observing action (sequences) performed by others [36]. Some accounts have explicitly made the point that premotor

activity can be understood as prediction of future extero- and interoceptive input caused by future movements, e.g. [37,38].

Section 3

Rostral anterior cingulate cortex (ACC)

If premotor areas encode dynamics over a time-scale of up to a few seconds, one might assume that the posterior part of rostral ACC (prACC), which lies rostrally to premotor areas, operates at an even slower time-scale. Many studies that report prACC activity use (learning) experiments, in which subjects make choices based on information presented in preceding trials; e.g., [39–41]. In these experiments, the effective time-scale of the representations (concepts) required for making decisions covers multiple trials, i.e., several seconds. In functional neuroimaging studies, prACC has been described as being involved in 'monitoring', 'decision making', 'conflict resolution' and 'updating of internal states'. Although these functions may sound incompatible, they all describe functions that entail operations on internal states, in a hierarchy, evolving at a slow time-scale, which cannot be reduced to short-term or instantaneous functions.

Furthermore, we speculate that the time-scales of representations in the rostral ACC depend on which part, in the rostro-caudal direction, is involved. For faster time-scales (seconds), we would expect more posterior locations (i.e., prACC) to be implicated. Conversely, more rostral locations may operate at slower time-scales. This hypothesis is consistent with a meta-analysis of neuroimaging studies involving the anterior part of the rostral ACC (arACC) [42]: Tasks involving 'person perception', 'mentalizing'[1], and 'self-knowledge' are attributed to arACC locations. All of these functions engage representations of an agent's (self or other) actions. We speculate that arACC encodes concepts that represent causal trajectories over extended periods and endow the representation of actions (self or other) with a context. For example, predictions about a friend's actions are constrained by conceptual representations of his/her intentions. It is

[1] Mentalizing refers to the cognitive process necessary to predict other people's behaviour in the future.

not the actions themselves that are represented in arACC, but the context that renders the action of oneself or others predictable.

Section 4

Lateral prefrontal cortex

There is a large literature on 'cognitive control' with respect to hierarchies in lateral prefrontal cortex. Three recent reviews summarise compelling findings that this hierarchy exhibits a rostro-caudal gradient [43–45]. Koechlin and Summerfield state that '…these data depict a hierarchically ordered executive system lying along the anterior-posterior axis of the lateral PFC, with control signals owing to events which occurred in the more and more distant past arising from successively more anterior cortical regions.' As noted in [43] by Badre: 'A recently popular hypothesis is that the rostro-caudal axis of prefrontal cortex supports a control hierarchy whereby posterior-to-anterior prefrontal cortex mediates progressively abstract, higher-order control.' And Botvinick in [44] states: '…, the prefrontal hierarchy is understood as involving levels of increasing temporal abstraction…'. In short, there are compelling perspectives and empirical findings [45–49] that support the hypothesis that the lateral prefrontal cortex is hierarchically structured according to temporal scale.

Section 5

Orbitofrontal cortex

Even more rostral to arACC lies the orbitofrontal cortex (OFC). The functionality of OFC is seemingly diverse. A short list contains: (i) signalling the affective value of stimuli (ii) encoding expectations of future reward, (iii) updating these expectations, (iv) contributing to decision making by using knowledge of the rules or structure of the decision problem [50]. In the following, we will sketch the idea how these functions can be expressed as operations on top-level representations in a cortical temporal hierarchy.

OFC as the top level represents the temporally most stable environmental states, namely rules [51,52]. Their stability over an extended period of time, with respect to the agent's

actions, affords decision making processes (at any level of the hierarchy) an advantage. This is because specific aspects of future sensory input can be inferred, far into the future, without having seen much data. Clearly, rules must be well-selected, otherwise decision making can go astray. Critically, a malfunction at the top level (OFC) has consequences that effect all temporal scales, because subordinate cortical levels attempt to explain environmental states without being guided by the appropriate high-level concept [53]. An important finding, from lesion and functional studies, is that orbitofrontal cortex supports dynamic switching between rules [54,55]. For example, in the 'reversal learning' task, subjects have to switch between opposing rules [56]. These rules are hidden from the subject and have to be inferred from preceding data trials. At first glance, dynamic switching between rules seems to contradict the notion that OFC encodes temporally stable rules (see below). However, switching between temporally stable rules is a hallmark of multistable, nonlinear hierarchical systems that adjudicate among competing models of the environmental context, e.g. [57]. Once there is sufficient evidence against one rule, orbitofrontal cortex may switch dynamically to a more appropriate rule which allows for better prediction of the sensory input. Here, 'evidence' can be used in a precise way, under our theoretical treatment, because the log-evidence is negative surprise; $\ln p(y(a) \mid m) \geq F$ and both are optimised under the free energy principle [10]. In other words, Eq. 5 describes exactly the neuronal dynamics that maximise the evidence for a particular model of environmental or experimental contingencies. Note that one can also observe dynamic switching between two slowly varying states in our simulations, see Fig. 3C. For surprising input it took the system some time to optimize the free energy. During this transient to a new (no input) state, the system exhibits a large prediction error. This transient rise in LFP activity can be interpreted as expression of switching from one dynamic state (perceiving a song) to another (no auditory input). This dynamic switching between slow representations might be an explanation why areas with slow time-scales (e.g., OFC) can rapidly switch between different stable concepts. This view of switching at 'event boundaries' is also supported by experimental findings in other domains, e.g., the auditory system or 'cognitive control' [47,58–60].

At a high level of the hierarchy conceptual inference will subsume many modality-specific representations. The representations in OFC may therefore provide predictions for several senses, particularly those that change relatively slowly such as interoceptive input. For example, when we are hungry, the effect that eating has on olfaction, gustation and interoceptive input is probably the same throughout adult life [61,62]. In other words, the way our body reacts to eating represents a causal trajectory that is itself stable and predictable. It is not the act of eating itself that OFC encodes, but the predictable changes in the internal milieu on eating. This line of reasoning might explain why 'decision making' studies find that OFC activity signals the 'value' of a sensory outcome (typically 'rewarding' food stimuli), while activity in rostral ACC (see above) represents the 'value' of an action [63–65].

Reference List

1. Ungerleider LG, Mishkin M (1982) Two cortical visual systems. In: D.J.Engle, M.A.Goodale, R.J.Mansfield, editors. Analysis of visual behaviour. Cambridge,MA: MIT Press. pp. 549-586.

2. Goodale MA, Westwood DA (2004) An evolving view of duplex vision: separate but interacting cortical pathways for perception and action. Curr Opin Neurobiol 14: 203-211.

3. Grill-Spector K (2003) The neural basis of object perception. Curr Opin Neurobiol 13: 159-166.

4. Rizzolatti G, Luppino G (2001) The cortical motor system. Neuron 31: 889-901.

5. Husain M, Nachev P (2007) Space and the parietal cortex. Trends Cogn Sci 11: 30-36.

6. Wandell BA, Dumoulin SO, Brewer AA (2007) Visual field maps in human cortex. Neuron 56: 366-383.

7. Kanwisher N, Yovel G (2006) The fusiform face area: a cortical region specialized for the perception of faces. Philos Trans R Soc Lond B Biol Sci 361: 2109-2128.

8. Khaytin I, Chen X, Royal DW, Ruiz O, Jermakowicz WJ, Siegel RM, Casagrande VA (2007) Functional Organization of Temporal Frequency Selectivity in Primate Visual Cortex. Cereb Cortex

9. Sun P, Ueno K, Waggoner RA, Gardner JL, Tanaka K, Cheng K (2007) A temporal frequency-dependent functional architecture in human V1 revealed by high-resolution fMRI. Nat Neurosci 10: 1404-1406.

10. Hasson U, Yang E, Vallines I, Heeger DJ, Rubin N (2008) A hierarchy of temporal receptive windows in human cortex. J Neurosci 28: 2539-2550.

11. Ghazanfar AA, Nicolelis MA (2001) Feature article: the structure and function of dynamic cortical and thalamic receptive fields. Cereb Cortex 11: 183-193.

12. Schultz J, Chuang L, Vuong QC (2008) A dynamic object-processing network: metric shape discrimination of dynamic objects by activation of occipitotemporal, parietal, and frontal cortices. Cereb Cortex 18: 1302-1313.

13. Konen CS, Kastner S (2008) Two hierarchically organized neural systems for object information in human visual cortex. Nat Neurosci 11: 224-231.

14. Giese MA, Poggio T (2003) Neural mechanisms for the recognition of biological movements. Nat Rev Neurosci 4: 179-192.

15. Huk AC, Shadlen MN (2005) Neural activity in macaque parietal cortex reflects temporal integration of visual motion signals during perceptual decision making. J Neurosci 25: 10420-10436.

16. Borra E, Belmalih A, Calzavara R, Gerbella M, Murata A, Rozzi S, Luppino G (2008) Cortical connections of the macaque anterior intraparietal (AIP) area. Cereb Cortex 18: 1094-1111.

17. Fogassi L, Luppino G (2005) Motor functions of the parietal lobe. Curr Opin Neurobiol 15: 626-631.

18. Hall DA, Johnsrude IS, Haggard MP, Palmer AR, Akeroyd MA, Summerfield AQ (2002) Spectral and temporal processing in human auditory cortex. Cereb Cortex 12: 140-149.

19. Patterson RD, Uppenkamp S, Johnsrude IS, Griffiths TD (2002) The processing of temporal pitch and melody information in auditory cortex. Neuron 36: 767-776.

20. Obleser J, Zimmermann J, Van Meter J, Rauschecker JP (2007) Multiple stages of auditory speech perception reflected in event-related FMRI. Cereb Cortex 17: 2251-2257.

21. Griffiths TD, Warren JD (2004) What is an auditory object? Nat Rev Neurosci 5: 887-892.

22. Poeppel D, Idsardi WJ, van W, V (2008) Speech perception at the interface of neurobiology and linguistics. Philos Trans R Soc Lond B Biol Sci 363: 1071-1086.

23. Rauschecker JP, Tian B (2000) Mechanisms and streams for processing of "what" and "where" in auditory cortex. Proc Natl Acad Sci U S A 97: 11800-11806.

24. Arnott SR, Binns MA, Grady CL, Alain C (2004) Assessing the auditory dual-pathway model in humans. Neuroimage 22: 401-408.

25. Friederici AD, Alter K (2004) Lateralization of auditory language functions: a dynamic dual pathway model. Brain Lang 89: 267-276.

26. Zatorre RJ, Belin P, Penhune VB (2002) Structure and function of auditory cortex: music and speech. Trends Cogn Sci 6: 37-46.

27. Boemio A, Fromm S, Braun A, Poeppel D (2005) Hierarchical and asymmetric temporal sensitivity in human auditory cortices. Nat Neurosci 8: 389-395.

28. Salmelin R, Schnitzler A, Parkkonen L, Biermann K, Helenius P, Kiviniemi K, Kuukka K, Schmitz F, Freund H (1999) Native language, gender, and functional organization of the auditory cortex. Proc Natl Acad Sci U S A 96: 10460-10465.

29. Ghazanfar AA, Krupa DJ, Nicolelis MA (2001) Role of cortical feedback in the receptive field structure and nonlinear response properties of somatosensory thalamic neurons. Exp Brain Res 141: 88-100.

30. Blankenburg F, Ruff CC, Deichmann R, Rees G, Driver J (2006) The cutaneous rabbit illusion affects human primary sensory cortex somatotopically. PLoS Biol 4: e69.

31. Schall JD, Thompson KG (1999) Neural selection and control of visually guided eye movements. Annu Rev Neurosci 22: 241-259.

32. Pandy MG (2001) Computer modeling and simulation of human movement. Annual Review of Biomedical Engineering 3: 245-273.

33. Paninski L, Fellows MR, Hatsopoulos NG, Donoghue JP (2004) Spatiotemporal tuning of motor cortical neurons for hand position and velocity. J Neurophysiol 91: 515-532.

34. Hatsopoulos NG, Xu Q, Amit Y (2007) Encoding of movement fragments in the motor cortex. J Neurosci 27: 5105-5114.

35. Hoshi E, Tanji J (2007) Distinctions between dorsal and ventral premotor areas: anatomical connectivity and functional properties. Curr Opin Neurobiol 17: 234-242.

36. Rizzolatti G, Craighero L (2004) The mirror-neuron system. Annu Rev Neurosci 27: 169-192.

37. Prinz W (2006) What re-enactment earns us. Cortex 42: 515-517.

38. Schubotz RI, von Cramon DY (2002) Predicting perceptual events activates corresponding motor schemes in lateral premotor cortex: an fMRI study. Neuroimage 15: 787-796.

39. Behrens TE, Woolrich MW, Walton ME, Rushworth MF (2007) Learning the value of information in an uncertain world. Nat Neurosci 10: 1214-1221.

40. Brown JW, Braver TS (2005) Learned predictions of error likelihood in the anterior cingulate cortex. Science 307: 1118-1121.

41. Ito S, Stuphorn V, Brown JW, Schall JD (2003) Performance monitoring by the anterior cingulate cortex during saccade countermanding. Science 302: 120-122.

42. Amodio DM, Frith CD (2006) Meeting of minds: the medial frontal cortex and social cognition. Nat Rev Neurosci 7: 268-277.

43. Badre D (2008) Cognitive control, hierarchy, and the rostro-caudal organization of the frontal lobes. Trends Cogn Sci 12: 193-200.

44. Botvinick MM (2008) Hierarchical models of behavior and prefrontal function. Trends Cogn Sci 12: 201-208.

45. Koechlin E, Summerfield C (2007) An information theoretical approach to prefrontal executive function. Trends Cogn Sci 11: 229-235.

46. Fuster JM (2001) The prefrontal cortex--an update: time is of the essence. Neuron 30: 319-333.

47. Koechlin E, Jubault T (2006) Broca's area and the hierarchical organization of human behavior. Neuron 50: 963-974.

48. Shima K, Isoda M, Mushiake H, Tanji J (2007) Categorization of behavioural sequences in the prefrontal cortex. Nature 445: 315-318.

49. Tanji J, Shima K, Mushiake H (2007) Concept-based behavioral planning and the lateral prefrontal cortex. Trends Cogn Sci 11: 528-534.

50. O'Doherty JP (2007) Lights, camembert, action! The role of human orbitofrontal cortex in encoding stimuli, rewards, and choices. Ann N Y Acad Sci 1121: 254-272.

51. Miller EK, Freedman DJ, Wallis JD (2002) The prefrontal cortex: categories, concepts and cognition. Philos Trans R Soc Lond B Biol Sci 357: 1123-1136.

52. Wallis JD, Anderson KC, Miller EK (2001) Single neurons in prefrontal cortex encode abstract rules. Nature 411: 953-956.

53. Bechara A, Tranel D, Damasio H (2000) Characterization of the decision-making deficit of patients with ventromedial prefrontal cortex lesions. Brain 123 (Pt 11): 2189-2202.

54. Fellows LK, Farah MJ (2005) Different underlying impairments in decision-making following ventromedial and dorsolateral frontal lobe damage in humans. Cereb Cortex 15: 58-63.

55. Hornak J, O'Doherty J, Bramham J, Rolls ET, Morris RG, Bullock PR, Polkey CE (2004) Reward-related reversal learning after surgical excisions in orbito-frontal or dorsolateral prefrontal cortex in humans. J Cogn Neurosci 16: 463-478.

56. Hampton AN, O'Doherty JP (2007) Decoding the neural substrates of reward-related decision making with functional MRI. Proc Natl Acad Sci U S A 104: 1377-1382.

57. Deco G, Rolls ET (2005) Synaptic and spiking dynamics underlying reward reversal in the orbitofrontal cortex. Cereb Cortex 15: 15-30.

58. Fujii N, Graybiel AM (2003) Representation of action sequence boundaries by macaque prefrontal cortical neurons. Science 301: 1246-1249.

59. Zacks JM, Braver TS, Sheridan MA, Donaldson DI, Snyder AZ, Ollinger JM, Buckner RL, Raichle ME (2001) Human brain activity time-locked to perceptual event boundaries. Nat Neurosci 4: 651-655.

60. Chait M, Poeppel D, de Cheveigne A, Simon JZ (2007) Processing asymmetry of transitions between order and disorder in human auditory cortex. J Neurosci 27: 5207-5214.

61. Gottfried JA, O'Doherty J, Dolan RJ (2003) Encoding predictive reward value in human amygdala and orbitofrontal cortex. Science 301: 1104-1107.

62. O'Doherty JP (2004) Reward representations and reward-related learning in the human brain: insights from neuroimaging. Curr Opin Neurobiol 14: 769-776.

63. Lee D, Rushworth MF, Walton ME, Watanabe M, Sakagami M (2007) Functional specialization of the primate frontal cortex during decision making. J Neurosci 27: 8170-8173.

64. Rushworth MF, Behrens TE, Rudebeck PH, Walton ME (2007) Contrasting roles for cingulate and orbitofrontal cortex in decisions and social behaviour. Trends Cogn Sci 11: 168-176.

65. Walton ME, Devlin JT, Rushworth MF (2004) Interactions between decision making and performance monitoring within prefrontal cortex. Nat Neurosci 7: 1259-1265.
